# Non-responsiveness to intravitreal aflibercept treatment in neovascular age-related macular degeneration: implications of serous pigment epithelial detachment

**DOI:** 10.1038/srep29619

**Published:** 2016-07-11

**Authors:** Norihiro Nagai, Misa Suzuki, Atsuro Uchida, Toshihide Kurihara, Mamoru Kamoshita, Sakiko Minami, Hajime Shinoda, Kazuo Tsubota, Yoko Ozawa

**Affiliations:** 1 Laboratory of Retinal Cell Biology, Keio University School of Medicine, 35 Shinanomachi, Shinjuku-ku, Tokyo 160-8582, Japan; 2 Department of Ophthalmology, Keio University School of Medicine, 35 Shinanomachi, Shinjuku-ku, Tokyo 160-8582, Japan.

## Abstract

The prognosis of neovascular age-related macular degeneration (AMD) has been improved by anti-vascular endothelial growth factor treatments, including intravitreal aflibercept (IVA) treatment. However, many patients remain incurable. In this study, we retrospectively evaluated non-responsiveness to IVA monotherapy at 12 months in 133 eyes of 133 AMD patients. Sixty-two patients were initially treatment-naive, and 71 had received other treatments before IVA (the treatment-switched group). Mean best-corrected visual acuity (BCVA) was improved in the treatment-naive group but not in the treatment-switched group, although mean central retinal thickness (CRT) decreased in both groups. The respective percentages of non-responders as determined by worsened BCVA in the treatment-naive and treatment-switched groups were 8.1% and 15.5%, and via fundus findings, they were 12.9% and 8.5%. Multivariate analyses adjusted for age, gender, CRT, and greatest linear dimension showed that serous pigment epithelial detachment (PED) at baseline was associated with non-responsiveness in both groups as determined by BCVA and by fundus findings, and fibrovascular PED measurements indicated no response as determined by fundus findings in the treatment-switched group. The results reported herein may assist the formulation of appropriate treatment protocols for AMD patients.

The incidence of age-related macular degeneration (AMD) is increasing in conjunction with an aging society^1^, and it is a leading cause of blindness worldwide^2^. Central visual impairment reduces the quality of life of otherwise healthy AMD patients and their carers^3^,^4^,^5^, therefore AMD constitutes a social issue. Recent research has determined that vascular endothelial growth factor (VEGF) is responsible for neovascular AMD, and three types of anti-VEGF drugs have been approved: pegaptanib^6^, ranibizumab^7^,^8^, and aflibercept^9^,^10^. These drugs have substantially improved the prognosis of neovascular AMD patients, but notably, not all patients have satisfactory outcomes, and non-responsiveness to treatment^11^,^12^ and tachyphylaxis to the drugs^13^,^14^ have recently been reported.

The third anti-VEGF drug to be approved, aflibercept, is a recombinant fusion protein consisting of VEGF binding portions of human VEGF receptors 1 and 2^15^. It binds not only to VEGF-A, but other VEGF family proteins, VEGF-B and placental growth factor (PlGF); in contrast to pegaptanib, which is an aptamer that binds to isoform 165 of VEGF-A, and ranibizumab, which is an affinity-enhanced humanized anti-VEGF-A antibody fragment. Aflibercept may suppress neovascularization through VEGF receptor 2 and exudative changes through VEGF receptor 1^15^. The results of two phase III studies (VEGF Trap-Eye: Investigation of Efficacy and Safety in Wet AMD [VIEW 1 and VIEW 2]) suggest that intravitreal aflibercept (IVA) treatment is not inferior to intravitreal ranibizumab (IVR) treatment with regard to clinical outcomes^9^,^10^. Moreover, recent reports show that IVA can successfully treat AMD patients who are not sufficiently responsive to IVR treatment^16^, and in some cases, IVA has succeeded in treating patients with serous pigment epithelial detachment (PED)^17^,^18^, which is one of the predictive factors of non-responsiveness to IVR treatment^11^. In a previous report, as many as 15–20% of treatment-naive AMD patients were non-responsive to IVR treatment, though the majority of the patients were responsive to IVR^11^. However, the potential efficacy of IVA treatment for AMD with or without previous treatment remains obscure.

In this study, we evaluated responsiveness to IVA treatment in treatment-naive patients and patients switched from other AMD treatments to IVA treatment. The clinical characteristics were analyzed at baseline. The results suggest that for apparent non-responders, it may be prudent to avoid the continuance of IVA treatment and to recommend to undertake other therapies.

## Results

### Baseline characteristics of patients

Treatment-naive and treatment-switched (patients who had received other treatments before IVA treatment) groups were composed of 62 eyes of 62 patients and 71 eyes of 71 patients, respectively. In the switched group, 53 eyes had been treated with a single type of anti-VEGF drug other than aflibercept; 48 eyes had been treated only with ranibizumab, 4 eyes had been treated only with pegaptanib, and 1 eye had been treated only with bevacizumab, which is an off-label use of the drug. Two eyes had been treated only with photodynamic therapy. The remaining 16 eyes had been treated with combinations of these therapies. On average, a total of 9.5 injections of anti-VEGF drugs, either ranibizumab, pegaptanib, or bevacizumab, were administered prior to the initial IVA treatment. There were no significant differences in age, gender, CRT, or greatest linear dimension (GLD) at baseline between the groups. The treatment-switched group included more type 1 choroidal neovascularization (CNV) (*p* = 0.0004) and fibrovascular PED (*p* = 0.017) than the treatment-naive group, and the treatment-naive group included more retinal hemorrhage than the treatment-switched group (*p* = 0.0006) (Table 1). The subtypes of wet AMD were defined according to the following official classifications and diagnostic criteria: Classification and Diagnostic Criteria of Age-Related Macular Degeneration^19^, Treatment Guidelines for Age-related Macular Degeneration of the Japanese Ophthalmological Society^20^, and Age-Related Macular Degeneration: Guidelines for Management of The Royal College of Ophthalmologists^21^.

### Changes in BCVA and CRT after 12 months of IVA treatment

**Figure 1:**
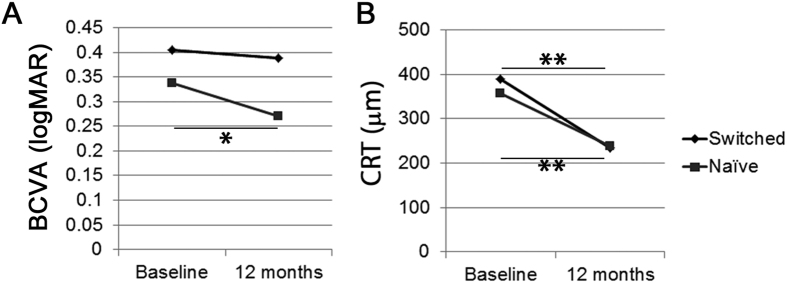
Mean best-corrected visual acuity (BCVA) and central retinal thickness (CRT) changes after intravitreal aflibercept (IVA) treatment at month 12. (**A**) Compared with baseline, mean BCVA was significantly improved in the treatment-naive group, but not in the treatment-switched group, while mean CRT was reduced in both the treatment-naive and the treatment-switched groups 12 months after IVA treatment. Two-tailed *t*-test, **p **<** 0.01.

The mean BCVA in the treatment-naive group, but not in the treatment-switched group, was significantly improved after 12 months of IVA treatment compared with baseline (Fig. 1A). IVA treatment significantly reduced the mean CRT in both groups at month 12 compared with baseline (Fig. 1B).

### Number of injections during IVA treatment

The mean numbers of injections were 5.1 ± 2.0 and 5.8 ± 1.8 in the treatment-naive and treatment-switched groups respectively, including the initial three injections and additional pro re nata (PRN) injections. The number of injections in the treatment-switched group was significantly more than that in the treatment-naive group (*p* = 0.03).

### Non-responders to IVA

We identified non-responders after IVA treatment either by worsened BCVA or fundus findings. BCVA-determined patients whose logMAR score worsened by more than 0.2 by month 12 compared with baseline were considered non-responders. The patients who had increased exudative fundus findings, such as increased intraretinal fluid, SRF, and hemorrhage, and enlarged PED, or increased CRT of more than 100 μm at month 12, or no decrease in CRT during treatment, both compared with baseline, were considered non-responders.

In the naive group, 5 eyes of 5 patients (8.1%) were non-responders as determined by BCVA, and 8 eyes of 8 patients (12.9%) were non-responders as determined by fundus findings (Table 2). In the treatment-switched group, 11 eyes of 11 patients (15.5%) were non-responders as determined by BCVA, and 6 eyes of 6 patients (8.5%) were non-responders as determined by fundus findings (Table 3). There were no significant differences between the ratio of non-responders in the treatment-naive and treatment-switched groups determined by either criterion. The demographics and baseline ocular characteristics of the non-responders in both groups are shown in Tables 2 and 3, respectively.

**Figure 2:**
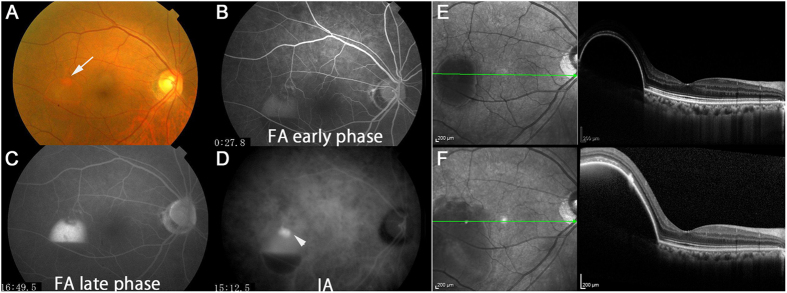
A non-responder as determined by fundus findings with serous pigment epithelial detachment (PED) in the treatment-naive group. This was a 72-year-old woman with treatment-naive polypoidal choroidal vasculopathy and best-corrected visual acuity (BCVA) of 1.0 in decimal (logMAR 0) at baseline. Consistent with the findings of the fundus color photograph (**A**, the arrow corresponds to polyp lesion) and fluorescein (**B**,**C**, early phase and late phase respectively) and indocyanine green (**D**, the arrowhead shows polyp lesion) angiograms, an optical coherence tomography image showed serous PED at baseline (**E**). Although BCVA did not change after 7 IVA injections, serous PED worsened at month 12 (**F**).

We compared the baseline characteristics of responders and non-responders, both in the treatment-naive (Table 4) and the treatment-switched (Table 5) groups using multivariate logistic regression models adjusted for age, gender, CRT, and GLD. Serous PED at baseline was associated with non-responsiveness as determined by both BCVA (OR 21.9, 95% CI 1.60–297), fundus findings (OR 30.0, 95% CI 2.60–346) in the treatment-naive group, and also in the treatment-switched group (BCVA, OR 5.14, 95% CI 1.16–22.8; fundus findings, OR 10.5, 95% CI 1.42–77.4). A representative case of a non-responder determined by fundus findings with serous PED at baseline in the treatment-naive group is shown in Fig. 2. Fibrovascular PED at baseline was associated with non-responsiveness as judged by fundus findings in the treatment-switched group (OR 13.4, 95% CI 1.33–134).

### Serous PED in the treatment-naive group

**Figure 3:**
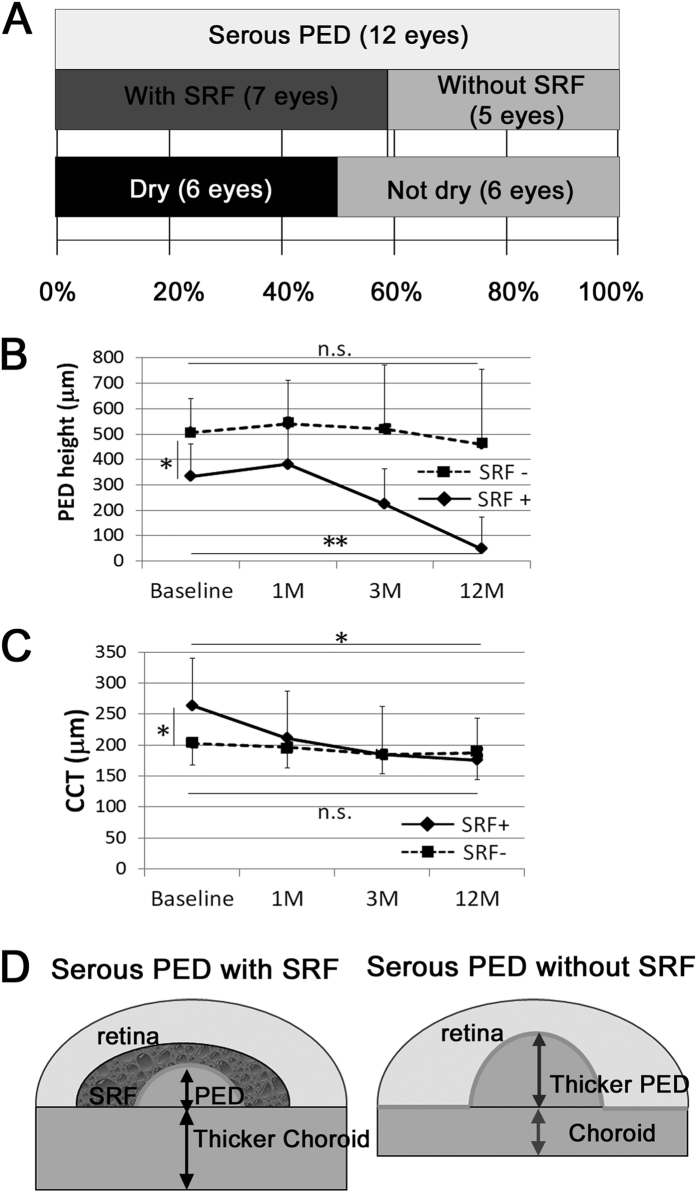
Detailed analyses of eyes with serous pigment epithelial detachment (PED) at baseline in the treatment-naive group. (**A**) Of 7 eyes with both serous PED and subretinal fluid (SRF) at baseline, 6 became dry at month 12. None of the 5 eyes which had serous PED but no SRF at baseline were dry at month 12. (**B,C**) PED height was smaller while CCT was greater in the eyes having serous PED with SRF at baseline. In the eyes having serous PED with SRF, mean PED height and CCT significantly decreased by IVA treatment at month 12. (**D**) Schematic models of serous PED with or without SRF. Two-tailed *t*-test, **p **<** 0.01, *p **<** 0.05.

**Figure 4:**
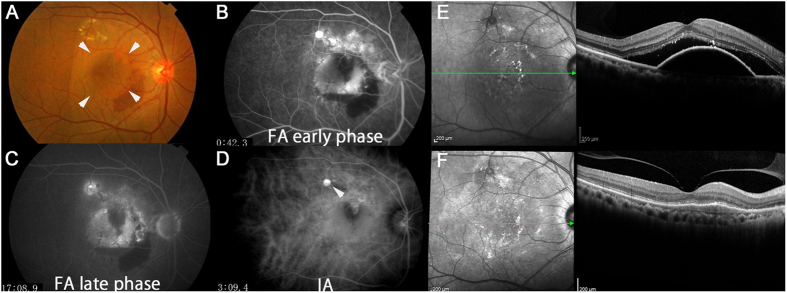
A representative case with serous pigment epithelial detachment (PED) and subretinal fluid (SRF) successfully treated with intravitreal aflibercept (IVA) in the treatment-naive group. This was a 63-year-old man with treatment-naive polypoidal choroidal vasculopathy and best-corrected visual acuity (BCVA) of 1.0 in decimal (logMAR 0) at baseline. Arrowheads in the fundus color photograph (**A**) indicate serous PED. Fluorescein (**B**,**C**, early phase and late phase respectively) and indocyanine green (**D**) angiograms. The arrowhead indicates a polyp lesion. An optical coherence tomography image (**E**) showed that at month 12, serous PED and SRF at baseline had disappeared after 7 IVA treatments.

Among the 12 eyes with serous PED at baseline, 6 eyes (50%) still had fluid, however the other 6 eyes (50%) exhibited no detectable serous PED in optical coherence tomography (OCT) images and had become dry by month 12 (Fig. 3A). Interestingly, all of the 6 eyes with no fluid at month 12, including intra- and subretinal fluid and serous PED, exhibited subretinal fluid (SRF) in addition to serous PED at baseline (Fig. 3A). The remaining 1 eye of the 7 eyes with both serous PED and SRF at baseline also exhibited a reduction in serous PED, although it had not become completely dry by month 12. Moreover, all 5 eyes with serous PED but no SRF at baseline did not become dry or exhibit a reduction in serous PED. Mean PED height was smaller and central choroidal thickness (CCT) was greater in the eyes having serous PED with SRF (Fig. 3B–D). Mean PED height decreased from 333 ± 128 μm at baseline to 47 ± 126 μm at 12 months (*p* = 0.001; percentage change from baseline, 14.3%) in the eyes having serous PED with SRF at baseline, while in the eyes having serous PED with no SRF at baseline, mean PED height (507 ± 132 μm) did not significantly change at 12 months (460 ± 295 μm [*p* = 0.82]). Mean CCT had a trend of decrease from 264 ± 77 μm at baseline to 185 ± 77 μm at 3 months (*p* = 0.07) and a significant decrease to 176 ± 68 μm at 12 months (*p* = 0.04; percentage change from baseline, 86.5%) in the eyes having serous PED with SRF at baseline, while in the eyes having serous PED with no SRF at baseline, mean CCT (203 ± 35 μm) did not significantly change afterwards compared with baseline (185 ± 31 μm [*p* = 0.40] and 188 ± 44 μm [*p* = 0.56], at 3 and 12 months, respectively). The numbers of injections in patients with serous PED with or without SRF at baseline were 5.7 ± 1.4 and 6.2 ± 1.8, respectively, and the difference between the two was not statistically significant (*p* = 0.60). A representative case that had serous PED with SRF at baseline and later became dry is shown in Fig. 4.

## Discussion

Aflibercept was the third anti-VEGF drug approved for AMD, which may reflect the relatively large number of patients switched to it from other treatments in this study. Most of the treatment-switched patients had been treated with several IVR injections before initial IVA injection, with an inadequacy or absence of treatment effects. Consistent with the fact that Type 1 CNV and fibrovascular PED are associated with risks of non-responsiveness to IVR^11^, these findings were observed more in the treatment-switched group before the initial IVA treatment.

The treatment-switched group showed improvement in CRT but not in BCVA, suggesting the possibility that the treatment-switched group had irreversible retinal neural dysfunction after a long course of pathological AMD. The treatment-switched group needed more IVA injections than the treatment-naive group, suggesting that the eyes were more obstinately resistant to anti-VEGF treatments.

There were 8.1% non-responders to IVA as determined by BCVA, and 12.9% as determined by fundus findings in this study, whereas in our previous study of treatment-naive AMD, 16.8% of the patients were non-responders to IVR as determined by BCVA and 21.0% were non-responders as determined by fundus findings^11^. These are separate studies and cannot be simply compared; however, the non-responders to IVA exhibited a trend of a smaller ratio. This could be related to aflibercept’s broader suppressive effects on VEGF family proteins, including VEGF-B and PlGF^22^, its greater affinity for VEGF-A^22^, and the longer half-life of the drug^22^. Alternatively, because progress in anti-VEGF therapy promoted early detection and treatment of AMD, the patients included in this IVA study may have had more recently developed lesions. This may account for the fact that only 3.2% of patients had fibrovascular PED in the treatment-naive group in the current study, in contrast to 32.0% in our previous IVR study^11^. The treatment naive patients in the current study had a mean age of 69.5 years and a mean BCVA of 0.34, while those in our previous IVR study had a mean age of 73.0 years and a mean BCVA of 0.41^11^, also suggesting that patients with more recently developed AMD might have been included in the treatment-naive group in this current study.

Because treatment effects are varied in AMD patients, personalized treatments are required^23^. To establish a definitive protocol for AMD treatment, research into single nucleotide polymorphisms (SNPs)^24^ related to the prognosis of AMD after anti-VEGF treatment is being actively performed^25^. However, AMD pathogenesis involves environmental factors^26^, resulting in a differential impact of clinical characteristics on responsiveness or non-responsiveness to treatment.

In the major clinical IVA studies, VIEW1 and 2 studies^9^, IVA injections were performed 3 times monthly in the dosing phase, followed by monthly or bi-monthly injections afterwards. In contrast, the current study used a PRN method following the 3 times monthly induction phase. While a CATT study showed that PRN treatment was equal to monthly treatment with ranibizumab^27^, there is no comparable study with aflibercept. Given that the VIEW1 and 2 studies^9^ showed non-inferiority of IVA to IVR, and given that the number of non-responders to IVA was smaller than the number of non-responders to IVR^11^, PRN treatment with IVA might also provide similar effects to monthly aflibercept treatments, although further study is required to investigate the potential similarity.

There are several previous reports describing the effects of IVA on serous PED^17^,^18^. In these reports, IVA was successful in the treatment of AMD patients who had serous PED. However, in the current study, serous PED was associated with resistance to IVA treatment. To clarify this apparent discrepancy, we reviewed the previous reports. One report involving 11 eyes that had been switched to IVA from the other treatments showed that 15 IVA injections in a PRN regimen over 18 months (thus, almost monthly injections) after a switch to IVA treatment reduced PED volume. However, the range of the mean reduction was only 20%^28^. In that report, it is not clear whether there were eyes in which PED disappeared or BCVA was improved. Another report showed that 8 treatment-naive eyes with serous PED treated with bi-monthly IVA after 3 monthly injections as a dosing phase showed that serous PED was reduced after each IVA treatment, but it recurred every time 1 month after the injection^17^, suggesting a limited effect of IVA on serous PED. During the current study, IVA injection was allowed to be performed whenever intra- or subretinal fluid was observed or PED was enlarged, thus treatment was performed at any time when exudative changes worsened. In fact, there was a trend of more injections being needed in the eyes with serous PED at baseline in the current study (serous PED 5.9 ± 1.5 vs. the others at 4.9 ± 2.1, *p* = 0.07). Nonetheless, serous PED was non-responsive to IVA determined by the BCVA and fundus finding criteria, indicating that AMD with serous PED was not easily treated by IVA.

Knowing that serous PED at baseline is a risk factor for non-responsiveness to IVA, we further investigated whether serous PED became dry with IVA treatment when it was accompanied by SRF. The affirmative results in this respect were consistent with a previous retrospective study involving IVR and IVA treatments, which showed that serous PED with SRF had a better visual prognosis, although the report did not include the incidence of resolution or details of the other fundus findings^18^. Interestingly, among the 6 eyes in 2 case reports with serous PED, all of which became dry after being switched to IVA treatment from the other treatment methods, 4 eyes had SRF before the first IVA treatment. Moreover, among these 6 eyes, 1 eye with rapid recurrence after exhibiting dryness by IVA treatment had no SRF at baseline. Taken together, the results suggest that serous PED with SRF may respond and become dry after IVA treatment.

The PED height was smaller while CCT was greater in the eyes having serous PED with SRF at baseline. However, the CCT was significantly reduced by IVA. It is generally accepted that IVA causes a reduction in CCT^29^,^30^, and that it is likely due to a reduction in the exudative activity of the choroid, inducing vasoconstriction and/or reducing choroidal fenestrations^31^. The underlying mechanism may involve the target molecules of aflibercept, namely VEGF-A, VEGF-B, PlGF, and a recently reported potential target, galectin-1^32^, which might have been more dominantly involved in the pathogenesis in the eyes having serous PED with SRF. Alternatively, the integrity of the retinal pigment epithelium (RPE) in the eyes with serous PED with SRF may be lower; therefore, the drug may have reached the sub-RPE space more easily, although further analyses are required to elucidate the relevant mechanism.

Fibrovascular PED was a predictive factor for non-responsiveness as determined by fundus findings in the treatment-switched group. This could be because RPE dysfunction due to the long-existing pathological condition might have developed before the initial IVA treatment.

The limitations of this study include that the sample size was relatively small, different AMD types were included among the study participants, and genetic analyses were not performed. Additionally, this was a retrospective study and not all the patients were examined every month, particularly in cases where the clinical course was progressing favorably.

In summary, IVA treatment was effective for most of the AMD patients; however, serous PED at baseline was a risk factor for non-responsiveness as determined by either BCVA or fundus findings at month 12, in both the treatment-naive and the treatment-switched groups. Fibrovascular PED at baseline was also associated with non-responsiveness as determined by fundus findings in the treatment-switched group. The current study will assist the understanding of the variable responsiveness of AMD to IVA treatment, and the deficiencies of AMD treatment that should be targeted in future studies to develop new therapeutic approaches.

## Methods

The study followed the tenets of the Declaration of Helsinki, was approved by the Ethics Committee of the Keio University School of Medicine (2010002), and was registered as UMIN000007649. Informed consent was obtained from all subjects.

This was a retrospective study based on detailed medical chart review. The study included 133 eyes of 133 patients with active neovascular AMD that were treated with IVA at the Medical Retina Division Clinic (AMD Clinic) of the Department of Ophthalmology, Keio University Hospital (Tokyo, Japan) between February 2012 and August 2014. Sixty-two patients were initially treatment-naive, and 71 patients had been receiving other treatments before IVA treatment (the treatment-switched group). All patients attended the clinic for at least 12 months, during which the only treatment they received was IVA.

All subjects underwent BCVA measurement with the refraction test throughout the course of treatment, slit-lamp examination, and binocular indirect ophthalmoscopy after pupil dilation with 0.5% tropicamide. These exams were performed at every follow-up visit.

Fluorescein angiography and indocyanine green (ICG) angiography were performed using a Topcon TRC50DX retinal Camera (Topcon, Tokyo, Japan) to diagnose AMD and determine its subtypes, typical AMD characterized by the choroidal neovascular membrane, polypoidal choroidal vasculopathy (PCV), and retinal angiomatous proliferation (RAP). Angiographic grading was conducted according to the TAP criteria by retina specialists in our clinic (NN, YO, MS, and TK)^33^. Using fluorescein angiography, significant PED was defined according to lesion diameter; serous PED > 2 disc diameters (DD), hemorrhagic PED > 2DD, and fibrovascular PED > 3DD.

OCT was used to evaluate CRT, intraretinal fluid, SRF accumulation, PED, and CCT. CRT was defined as the distance between the internal limiting membrane and the presumed RPE at the fovea. OCT was performed at every follow-up visit using a Heidelberg Spectralis OCT (Heidelberg Engineering, Dossenheim, Germany) instrument. Measurement was performed by referring to the scale bars in the OCT system.

Aflibercept (2 mg, 0.05 mL) was injected intravitreally under sterile conditions via the pars plana once a month for 3 months, as an induction phase. Re-injections were given if the OCT image and/or fundus examination showed evidence of any exudative changes in the macula, identified as macular edema or SRF, or enlargement of a PED, or hemorrhage, at the time of follow-up examinations. Follow-up was carried out every month, but when exudative changes including intra- and/or subretinal and/or sub-RPE fluid, and/or hemorrhage were absent for more than 2 months, the interval was extended to up to 2 months. Any new subretinal or intraretinal hemorrhage or unexplained visual loss of more than 0.2 (logMAR score), most likely due to the undetected changes in the fundus findings and/or OCT images of the foveal section at the time of visits, was also treated.

Commercially available software (SPSS, V.23.0) was used for the statistical analysis. The demographic characteristics of the responders and non-responders were compared using the two-tailed *t*-test, and statistical significance was defined as *p* **<** 0.05. In the multivariable logistic regression analyses, potential risk factors for non-responders were adjusted for age, gender, CRT, and GLD at the time of initial IVA.

## Additional Information

**How to cite this article**: Nagai, N. *et al*. Non-responsiveness to intravitreal aflibercept treatment in neovascular age-related macular degeneration: implications of serous pigment epithelial detachment. *Sci. Rep*. **6**, 29619; doi: 10.1038/srep29619 (2016).

## Figures and Tables

**Table 1:** Baseline characteristics.

	Treatment-naive	Treatment-switched	*P*
Eyes	62	71	
Age, mean ± SD	69.5 ± 10.2	72.0 ± 8.5	0.127
Male, no. eyes (male %)	41 (66.1)	51 (71.8)	0.477
AMD type			0.648
Typical AMD (%)	23 (37.1)	32 (45.1)	
PCV (%)	37 (59.7)	37 (52.1)	
RAP (%)	2 (3.2)	2 (2.8)	
BCVA, mean ± SD (logMAR)	0.34 ± 0.40	0.40 ± 0.38	0.440
CRT, mean ± SD (μm)	358 ± 191	389 ± 215	0.380
GLD, mean ± SD (μm)	4298 ± 2591	4270 ± 2522	0.949
CCT, mean ± SD (μm)	204 ± 64	200 ± 60	0.093
Type 1 CNV (eyes [%])	17 (27.4)	41 (57.7)	*0.0004
Fundus findings, (eyes [%])			
Serous PED (>2DD)	12 (19.4)	10 (14.1)	0.414
Fibrovascular PED (>3DD)	2 (3.2)	11 (15.4)	*0.017
Hemorrhagic PED (>3DD)	6 (11.2)	2 (2.8)	0.097
Serous retinal detachment	48 (77.4)	57 (80.3)	0.686
Macular edema	14 (22.6)	16 (22.5)	0.995
Retinal hemorrhage	26 (41.9)	10 (14.1)	*0.0006

AMD, age-related macular degeneration; PCV, polypoidal choroidal vasculopathy; RAP, retinal angiomatous proliferation; BCVA, best corrected visual acuity; CNV, choroidal neovascularization; CRT, central retinal thickness; GLD, greatest linear dimension; CCT, central choroidal thickness; PED, pigment epithelial detachment. Two-tailed *t*-test, *p < 0.05, **p < 0.01.

**Table 2:** Demographics and baseline ocular characteristics of treatment-naive patients.

	BCVA responders	BCVA non-responders	Fundus responders	Fundus non-responders
	(57 eyes)	(5 eyes)	(54 eyes)	(8 eyes)
Ratio to naive patients (%)	91.9	8.1	87.1	12.9
Age, mean ± SD	68.9 ± 10.2	75.8 ± 7.8	70.0 ± 10.5	66.3 ± 7.6
Male, (eyes [%])	39 (68.4)	2 (40.0)	37 (68.5)	4 (50.0)
AMD type				
Typical AMD	21	2	21	2
PCV	35	2	31	6
RAP	1	1	2	0
BCVA, mean ± SD (logMAR)	0.34 ± 0.41	0.30 ± 0.23	0.37 ± 0.42	0.11 ± 0.23**
CRT, mean ± SD (μm)	364 ± 196	283 ± 133	355 ± 191	371 ± 209
GLD, mean ± SD (μm)	4185 ± 2608	5588 ± 2202	4307 ± 2681	4236 ± 2011
CCT, mean ± SD (μm)	204 ± 64	200 ± 60	203 ± 64	212 ± 58
Type 1 CNV (eyes [%])	15 (26.3)	2 (40.0)	15 (28.8)	2 (25.0)

AMD, age-related macular degeneration; PCV, polypoidal choroidal vasculopathy; RAP, retinal angiomatous proliferation; BCVA, best corrected visual acuity; CNV, choroidal neovascularization; CRT, central retinal thickness; GLD, greatest linear dimension; CCT, central choroidal thickness. Two-tailed *t*-test, **p < 0.01.

**Table 3:** Demographics and initial ocular characteristics of treatment-switched patients.

	BCVA responders	BCVA non-responders	Fundus responders	Fundus non-responders
	(60 eyes)	(11 eyes)	(65 eyes)	(6 eyes)
Ratio to switched patients (%)	84.5	15.5	91.5	8.5
Age, mean ± SD	71.8 ± 8.7	73.4 ± 7.7	72.2 ± 8.5	69.3 ± 8.3
Male, (eyes [%[)	44 (73.3)	7 (63.6)	46 (70.8)	5 (83.3)
AMD type				
Typical AMD	26	6	29	3
PCV	32	5	34	3
RAP	2	0	2	0
BCVA, mean ± SD (logMAR)	0.39 ± 0.39	0.48 ± 0.30	0.42 ± 0.39	0.18 ± 0.21
CRT, mean ± SD (μm)	379 ± 218	446 ± 195	393 ± 216	346 ± 212
GLD, mean ± SD (μm)	4111 ± 2341	5131 ± 3344	4385 ± 2556	3021 ± 1831
CCT, mean ± SD (μm)	185 ± 55	191 ± 60	190 ± 48	186 ± 56
Type 1 CNV (eyes [%])	36 (60.0)	5 (45.5)	38 (58.4)	3 (50.0)

AMD, age-related macular degeneration; PCV, polypoidal choroidal vasculopathy; RAP, retinal angiomatous proliferation; BCVA, best corrected visual acuity; CNV, choroidal neovascularization; CRT, central retinal thickness; GLD, greatest linear dimension; CCT, central choroidal thickness.

**Table 4:** Predictors of non-responders after intravitreal aflibercept therapy for treatment-naive patients.

	BCVA			Fundus finding		
	p value	OR	95%CI	p value	OR	95%CI
BCVA	0.514	0.355	0.016–7.974	0.806	1.000	1.000–1.000
CCT	0.756	1.003	0.985–1.021	0.604	1.003	0.990–1.017
AMD type						
Typical AMD	0.841	1.261	0.130–12.22	0.483	0.515	0.081–3.283
PCV	0.424	0.359	0.029–4.411	0.306	2.675	0.406–17.64
Type 1 CNV	0.439	1.551	0.180–13.34	0.991	1.010	0.174–5.849
Fundus findings						
serous PED (>2DD)	0.021*	21.873	1.597–297.1	0.006**	29.98	2.599–345.8
fibrovascular PED (>3DD)	–^†^	–^†^	–^†^	–^†^	–^†^	–^†^
subretinal fluid	0.780	0.706	0.061–8.178	0.088	0.240	0.046–1.239
macular edema	0.258	3.713	0.382–36.12	0.556	0.507	0.053–4.869
retinal hemorrhage	0.867	1.225	0.114–13.21	0.258	0.329	0.048–2.255

Multivariable logistic regression analyses adjusted for age, gender, CRT, and GLD at the time of initial IVA. Serous PED > 2 DD and fibrovascular PED > 3 DD were included. *p** < **0.05, **p** < **0.01.

^†^Unanalyzable because all subjects were responders.

BCVA, best corrected visual acuity; CCT, central choroidal thickness; AMD, age-related macular degeneration; PCV, polypoidal choroidal vasculopathy; CNV, choroidal neovascularization; PED, pigment epithelial detachment; CRT, central retinal thickness; GLD, greatest linear dimension.

**Table 5:** Predictors of non-responders after intravitreal aflibercept therapy for treatment-switched patients.

	**BCVA**			**Fundus finding**		
	**p value**	**OR**	**95%CI**	**p value**	**OR**	**95%CI**
BCVA	0.844	1.233	0.154–9.837	0.131	0.071	0.002–2.202
CCT	0.542	1.004	0.992–1.016	0.987	1.000	0.984–1.014
AMD type						
tAMD	0.305	2.174	0.493–9.585	0.739	0.756	0.146–3.921
PCV	0.559	0.127	0.127–2.463	0.664	1.444	0.275–7.586
Type 1 NCV	0.504	0.626	0.158–2.480	0.769	0.783	0.153–4.004
Fundus findings						
serous PED (>2DD)	0.031*	5.143	1.159–22.82	0.021*	10.466	1.415–77.42
fibrovascular PED (>3DD)	0.465	0.433	0.046–4.097	0.028*	13.36	1.328–134.4
subretinal fluid	0.999	0.000		0.407	0.451	0.069–2.959
macular edema	0.207	0.237	0.025–2.224	0.998	0.000	
retinal hemorrhage	0.661	0.610	0.067–5.574	0.714	1.581	0.137–18.26

Multivariable logistic regression analyses adjusted for age, gender, CRT, and GLD at the time of initial IVA. Serous PED > 2 DD and fibrovascular PED > 3 DD were included. *p** < **0.05.

BCVA, best corrected visual acuity; CCT, central choroidal thickness; AMD, age-related macular degeneration; PCV, polypoidal choroidal vasculopathy; CNV, choroidal neovascularization; PED, pigment epithelial detachment; CRT, central retinal thickness; GLD, greatest linear dimension.
